# Vascular endothelial growth factor and the risk of venous thromboembolism: a genetic correlation and two-sample Mendelian randomization study

**DOI:** 10.1186/s12959-022-00427-6

**Published:** 2022-11-08

**Authors:** Qiaoyun Zhang, Xiaoyu Zhang, Jie Zhang, Biyan Wang, Qiuyue Tian, Xiaoni Meng, Jinxia Zhang, Mengyang Jiang, Yiqiang Zhang, Deqiang Zheng, Lijuan Wu, Wei Wang, Baoguo Wang, Youxin Wang

**Affiliations:** 1 School of Public Health, Capital Medical University, No. 10 Xitoutiao, Youanmenwai Street, Fengtai District, 100069 Beijing, China; 2 Department of Anesthesiology, Beijing Sanbo Brain Hospital, Capital Medical University, 50 Yikesong Road, Haidian District, 100093 Beijing, China; 3 Beijing Key Laboratory of Clinical Epidemiology, School of Public Health, Capital Medical University, Beijing, China; 4 Centre for Precision Medicine, Edith Cowan University, Joondalup, WA Australia

**Keywords:** Vascular endothelial growth factor, Venous thromboembolism, Mendelian randomization, Genetic correlation

## Abstract

**Background:**

The relationship between vascular endothelial growth factor (VEGF) and the risk of venous thromboembolism (VTE) has always been one of the concerns in the medical field. However, the causal inferences from published observational studies on this issue may be affected by confounders or reverse causality. We performed a two-sample bidirectional Mendelian randomization (MR) to infer the associations between VEGF and VTE.

**Methods:**

Summary statistics from genome-wide association studies (GWAS) for VEGF and VTE were obtained from published meta-analysis studies and the FinnGen consortium, respectively. Independent genetic variables significantly associated with exposure were selected as instrumental variables. Linkage disequilibrium score regression (LDSC) and five robust MR analytical approaches were conducted to estimate the genetic correlations and causal inference. The MR-Egger intercept, Cochran’s Q, and MR pleiotropy residual sum and outlier (MR-PRESSO) were performed to evaluate the horizontal pleiotropy, heterogeneities, and stability of these genetic variants on outcomes. Notably, replication analyses were performed using different subgroups of VTE.

**Results:**

LDSC failed to identify genetic correlations between VEGF and VTE. Based on 9 SNPs, the circulating VEGF level was positively related to the risk of VTE using inverse variance weighting (IVW) method (odds ratio (OR) = 1.064, 95% confidence interval (CI), 1.009–1.122). Reverse MR analyses showed that genetic liability for VTE was not associated with increased VEGF level (β = -0.021, 95% CI, -0.087-0.045). Pleiotropy-robust methods indicated no bias in any estimates.

**Conclusions:**

Our findings failed to detect coheritability between VEGF and VTE. The suggestive positive effect of the higher VEGF level on the VTE risk may have clinical implications, suggesting that VEGF as a possible predictor and therapeutic target for VTE prevention need to be further warranted.

**Supplementary Information:**

The online version contains supplementary material available at 10.1186/s12959-022-00427-6.

## Background

Venous thromboembolism (VTE) is a chronic disease that can be divided into deep vein thrombosis (DVT) and pulmonary embolism (PE) according to the site of embolism, and is the third leading cause of vascular mortality, affecting nearly 10 million population worldwide each year [1, 2]. VTE is a multicausal disorder influenced by both acquired and inherited risk factors, and is associated with reduced survival, high recurrence rates, and substantial healthcare costs [3–5]. Previous studies have focused on clinical risk factors (cancer, major surgery, immobilization, etc.) and some specific genetic conditions (i.e. Factor V, protein C or protein S) that account for less than one-fifth of population attributable risk in the elderly [6, 7], but most VTE are provoked by weak risk factors or even no apparent risk factors [1, 8]. Given the lack of public awareness that unprovoked thrombus is common and preventable as well as the few reliable and sensitive biomarkers to identify those patients, new biomarkers are still needed to alleviate cost and time of diagnosis [9]. One such possible biomarker is vascular endothelial growth factor (VEGF), a neurotrophic and angiogenic factor secreted by endothelial cells, which is known to affect a variety of physiological and pathological processes [10, 11]. Observational studies highlight the role of VEGF as an inflammatory marker in thrombosis [12, 13]. Besides, the formation of DVT may also stimulate the expression of VEGF [14]. However, inconsistent conclusions were also existed, such as the use of VEGF-inhibitors did not increase the risk of VTE in patients with ovarian cancer [15], but significantly increased the risk of VTE in patients with malignant glioma [16]. Therefore, whether there are causal associations of VEGF with risk of VTE require further investigations as the potential confounders as well as the above conflicting results.

The design of Mendelian randomization (MR) study follows Mendelian law of inheritance, which is similar to randomized controlled trails and could provide more robust evidence for causal estimation between VEGF and VTE risk. Genetic variants robustly related to VEGF and VTE would be selected as instrumental variables (IVs), respectively. IVs are less likely to be influenced by confounders and reverse causality due to the random assignment of parents to offspring at conception [17, 18]. Linkage disequilibrium score (LDSC) regression was performed to explore the coheritability of VEGF with VTE by assessing the genetic correlation [19]. This study was focus on the circulating VEGF, a possible regulator influencing the risk of VTE, the correlations of which with risk of VTE are not yet well defined [20]. Therefore, we applied the univariable MR (UVMR) and bidirectional MR analyses to infer causal association of circulating VEGF level with the risk of VTE using summary GWAS data from European population.

## Methods

### Study Design

**Fig. 1:**
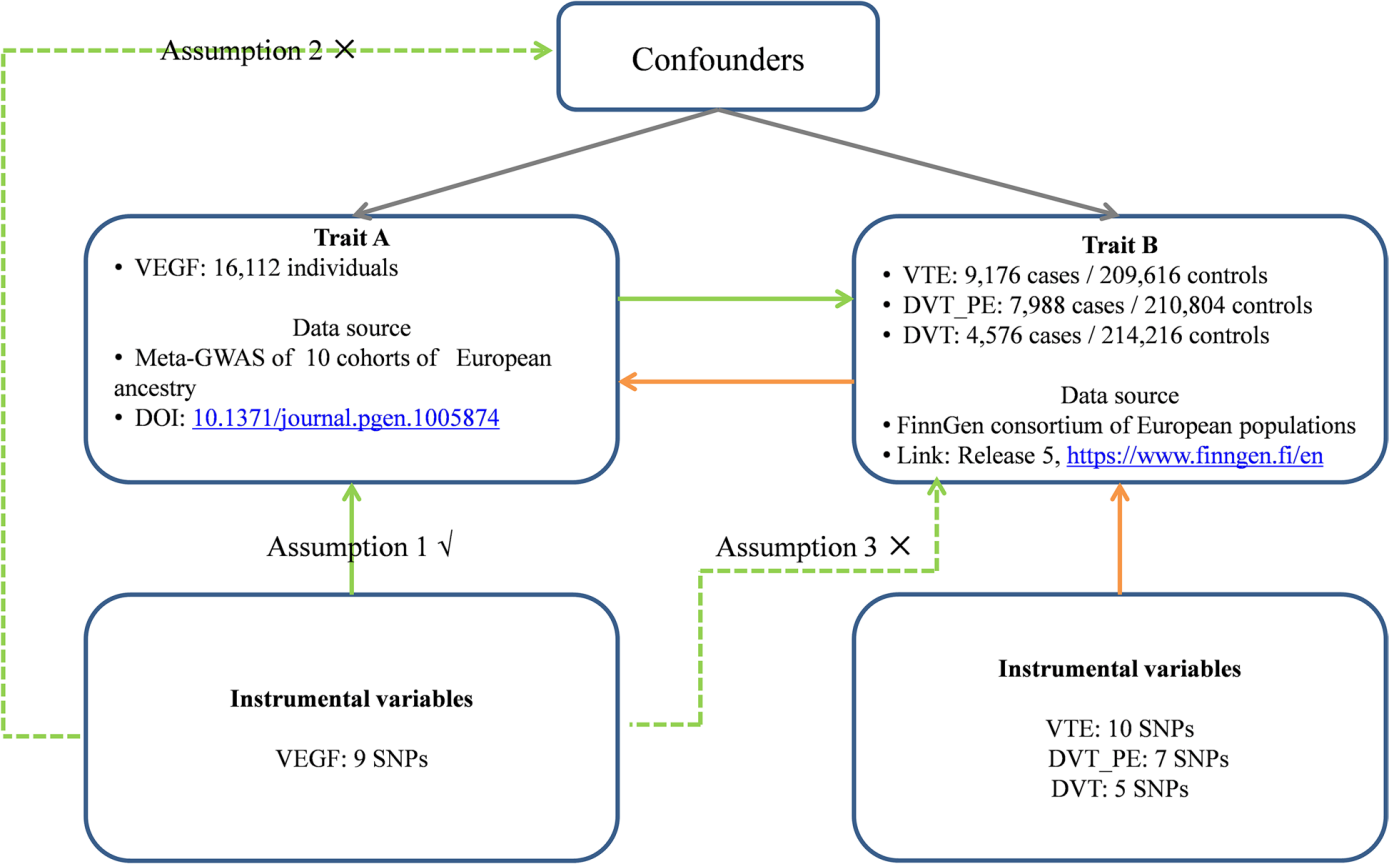
Schematic diagram of the study design. The dashed lines represent pathways that violate the MR assumptions. SNP: single-nucleotide polymorphism; GWAS: genome-wide association study; VEGF: vascular endothelial growth factor; VTE: venous thromboembolism; DVT: deep vein thrombosis; DVT_PE: deep vein thrombosis and pulmonary embolism

This is a two-sample bidirectional MR study. The genetic variants significantly related to VEGF and VTE were selected as instrumental variants, respectively. Schematic diagram of the study design and three major assumptions of forward MR are shown in Fig. 1. First, the IVs should be strongly correlated with VEGF. Second, the IVs have no associations with confounders. Last, the IVs should only be linked with VTE via VEGF. VTE (incidence rate: 115–269/100,000 persons/year [3]) includes DVT (incidence rate: 88–112/100,000 persons/year [7]) and PE. The lower extremities are the most common site for DVT, while PE occurs in pulmonary arteries, when thrombi dislodge from the vein walls and travel with the blood into the pulmonary arteries [21]. Therefore, replication analyses were performed using different subgroups of VTE (DVT_PE: DVT of the lower extremities and pulmonary embolism and DVT: DVT of the lower extremities (no controls excluded)). All statistical analyses in our study were based on available summary data and therefore no ethical approval was required.

### GWAS of VEGF

The summary data of VEGF were derived from the largest published GWAS meta-analysis based on 16,112 individuals (mean age: 54.8 years, 54% females) from ten cohorts of European ancestry. Of these, six cohorts (the Age Gene/Environment Susceptibility Reykjavik Study (AGES), the Cilento study (Cilento), the Framingham Heart Study (FHS), the Ogliastra Genetic Park (OGP), the Prospective Investigation of the Vasculature in Uppsala Seniors Study (PIVUS), and the Val Borbera study (VB)) were used as discovery datasets, another two cohorts (the Gioi and the Sorbs population) were served as discovery and in-silico replication cohorts, and the last two cohorts (the STANISLAS Family Study (SFS) and a sample of hypertensive adults (HT)) were served as discovery, in-silico and de-novo replication cohorts. In this association analyses, age and gender were adjusted as covariates. Ultimately, a total of 10 independent variants located in 7 chromosomal loci were identified in this GWAS meta-analysis of circulating VEGF levels. The unit of VEGF level was pg/ml and was natural log-transformed, other details was provided elsewhere [11].

### GWAS of VTE (DVT_PE and DVT)

We used summary statistic from a GWAS which was made public by the FinnGen consortium [22] (Release 5, https://www.finngen.fi/en). FinnGen is a growing project, containing many biobanks and cohorts (e.g., Auria Biobank, Biobank Borealis of Northern Finland, Finnish Clinical Biobank Tampere). A total of 218,792 samples and 16,962,023 variants were used for core analysis, with sex, age, and genotyping batch adjusted in this model. VTE, DVT_PE and DVT were defined according to the International Classification of Diseases (ICD) revision 9. VTE, the primary outcome, included 9,176 patients and 209,616 controls (https://storage.googleapis.com/finngen-public-data-r5/summary_stats/finngen_R5_I9_VTE.gz). Replication analyses were performed using 7,988 patients and 210,804 controls for DVT of the lower extremities and pulmonary embolism (https://storage.googleapis.com/finngen-public-data-r5/summary_stats/finngen_R5_I9_DVTANDPULM.gz), and 4,576 patients and 214,216 controls for DVT of the lower extremities (no controls excluded) (https://storage.googleapis.com/finngen-public-data-r5/summary_stats/finngen_R5_I9_PHLETHROMBDVTLOW_EXNONE.gz).

The GWAS summary data on exposure and outcomes were all based on European populations.

### Genetic instrumental variable selection

Single nucleotide polymorphisms (SNPs), that significantly (*P* threshold = 5 × 10^− 8^) associated with the circulating VEGF level and VTE risk, were selected as instrumental variables, respectively. Independent variants (linkage disequilibrium (LD), *r*^2^ < 0.001) were retained based on European ancestry reference data from the 1000 Genomes Project. Since the number of independent SNPs of VEGF was limited (only 3 SNPs retained after harmonizing VEGF with VTE), referring to similar studies that set r^2^ to 0.01 [23–25], we selected eligible SNPs by relaxing the LD r^2^ threshold to 0.01 when it was treated as exposure.

### MR analysis

Forward MR analyses were performed to estimate the causal effect of circulation VEGF (exposure) on the risk of VTE (DVT_PE and DVT) (outcomes). Then, reverse MR was conducted using genetic variants with VTE (DVT_PE and DVT) (exposures) respectively to investigate their causal effects on VEGF (outcome). The effects (i.e. beta) and corresponding standard errors (SE) of SNPs were obtained from the GWAS-VEGF and GWAS-VTE [26]. Next, palindromic SNPs were removed via harmonizing VEGF and VTE data [27].

Inverse variance-weighted (IVW) analysis was performed as the main method, actually that was a single variable weighted linear regression of outcome (SNPs) effects on exposure (SNPs) effects and the intercept was constrained to zero [28]. Results may be imprecise if IVs exhibit horizontal pleiotropy, meaning that IVs may affect outcomes via pathways other than exposures [29]. Therefore, we supplementarily applied several MR methods based on different IVs assumption, including MR-Egger regression, weighted median (WM), penalty weighted median (PWM) and causal analysis using summary effect estimates (CAUSE), as sensitivity analyses to verify the robustness of the main IVW estimate [29]. The MR-Egger regression (the intercept is not constrained to zero [29, 30]) gives consistent estimates with IVW method if all IVs are invalid; while WM and PWM methods require more than half of the IVs to be valid [31]. For efficiency, WM estimates are generally as accurate as IVW estimates, both are more accurate than MR-Egger estimates, and MR-Egger regression estimates are especially imprecise if IVs are all similarly associated with the exposure [31]. Horizontal pleiotropy may be correlated (IVs affect exposure and outcome through shared factors) or not correlated (IVs affect exposure and outcome via independent pathways) with a shared factor but both of which are not violated the major MR assumption [32]. The CAUSE analysis, a recent method that accounts for correlated or uncorrelated horizontal pleiotropy effects, was conducted, which includes more IVs by LD pruning (*r*^2^ < 0.10) with its built-in function based on pre-computed LD estimates [32].

Horizontal pleiotropy was assessed by the intercept test of MR-Egger method (the intercept *p*-value < 0.05 implied the presence of horizontal pleiotropy) [33] and the MR pleiotropy residual sum and outlier (MR-PRESSO) test (potential outlier SNPs which violated of the IV assumptions could be detected) [34]. In addition, heterogeneity was estimated by Cochran *Q* test and *I*^*2*^ statistics in IVW and MR-Egger methods (the Cochran *Q*_*P* value < 0.05 or *I*^*2*^ statistics > 25% indicated the presence of heterogeneity) [35, 36], which could help to evaluate the horizontal pleiotropy. Funnel plots were also used to assess potential asymmetry visually [29].

Odds ratios (ORs) and the corresponding 95% confidence intervals (CIs) of VTE correspond to VTE risk per standard deviation (SD) increase in log odds of the circulating VEGF, alternatively, β and the corresponding 95% CI of VEGF represent the reverse association. Bonferroni correction was performed to account for 3 outcomes, with the significance threshold for forward and reverse causality set at *P* = 0.017 (0.05/3). Referring to previous articles [37–39], we considered the results to be strong significant when *P* < 0.017, and suggestive evidence when 0.017 < *P* < 0.05. MR analyses were conducted by using the following R (version 4.0.3, https://www.r-project.org/) packages: “TwoSampleMR” [40, 41], “MR-PRESSO” [42] and “CAUSE” [32].

### Variance explained by IVs and F-statistic of MR analyses

To estimate the variance explained for each SNP, we calculated R^2^ by formula as follow: *R*^2^ = 2×MAF× (1-MAF) × Beta^2^. Then, we summed the R^2^ to calculate the overall R^2^ and F-statistics for exposure (F-statistic = R^2^ × (N-2) / (1-R^2^)). N means the number of individuals of the GWAS- exposure [43]. The higher the R^2^ and F-statistics are, the lower the risk of weak IVs bias [44].

### Heritability and genetic correlations analyses

LDSC regression regressed Chi-square statistics for one trait to calculate SNP-based heritability (h^2^) or two traits to estimate SNP-based coheritability (http://ldsc.broadinstitute.org/ldhub/, LD score tool, version 1.0.1). Cross-trait LDSC regression was conducted to assess the genetic correlations between VEGF and VTE by the regression slope using GWAS summary data [19]. If the heritability z-score is small (i.e. < 4), the genetic correlation estimates are generally too noisy to report [45]. Likewise, the results are probably not suitable for LDSC regression with small Chi-square (e.g., < 1.020) [46].

## Results

The detailed information for the characteristics of SNPs used for each trait was shown in supplementary material (Table S1 and Table S2). The brief information of GWAS data were listed in Table 1.

**Table 1:** Brief information of GWAS used in the MR analyses

Exposure		Outcome				
Trait	Sample	Trait	Sample	nIVs	R^2^	F-statistic
VEGF (r^2^ < 0.01)	16,112	VTE	218,792	9	0.166	39967.866
VTE (r^2^ < 0.001)	218,792	VEGF	16,112	10	0.130	29895.544
DVT_PE (r^2^ < 0.001)	218,792	VEGF	16,112	7	0.125	28358.117
DVT (r^2^ < 0.001)	218,792	VEGF	16,112	5	0.177	42556.074

*VEGF* Vascular endothelial growth factor, *VTE* Venous thromboembolism, *DVT* Deep vein thrombosis, *DVT_PE* Deep vein thrombosis and pulmonary embolism, *MR* Mendelian randomization, *R*^*2*^ Variance for SNPs; *nIVs* Number of instrumental variables

### Causal effect of VEGF on the risk of VTE via forward MR

In the forward MR analyses, a total of 11 SNPs were screened out and the F-statistics ranged from 291 to 29,703. Nine independent SNPs (rs7030781 and rs10761731 were excluded for being palindromic structure) were selected as the IVs for VEGF after harmonizing SNP-exposure and SNP-outcomes.

**Fig. 2:**
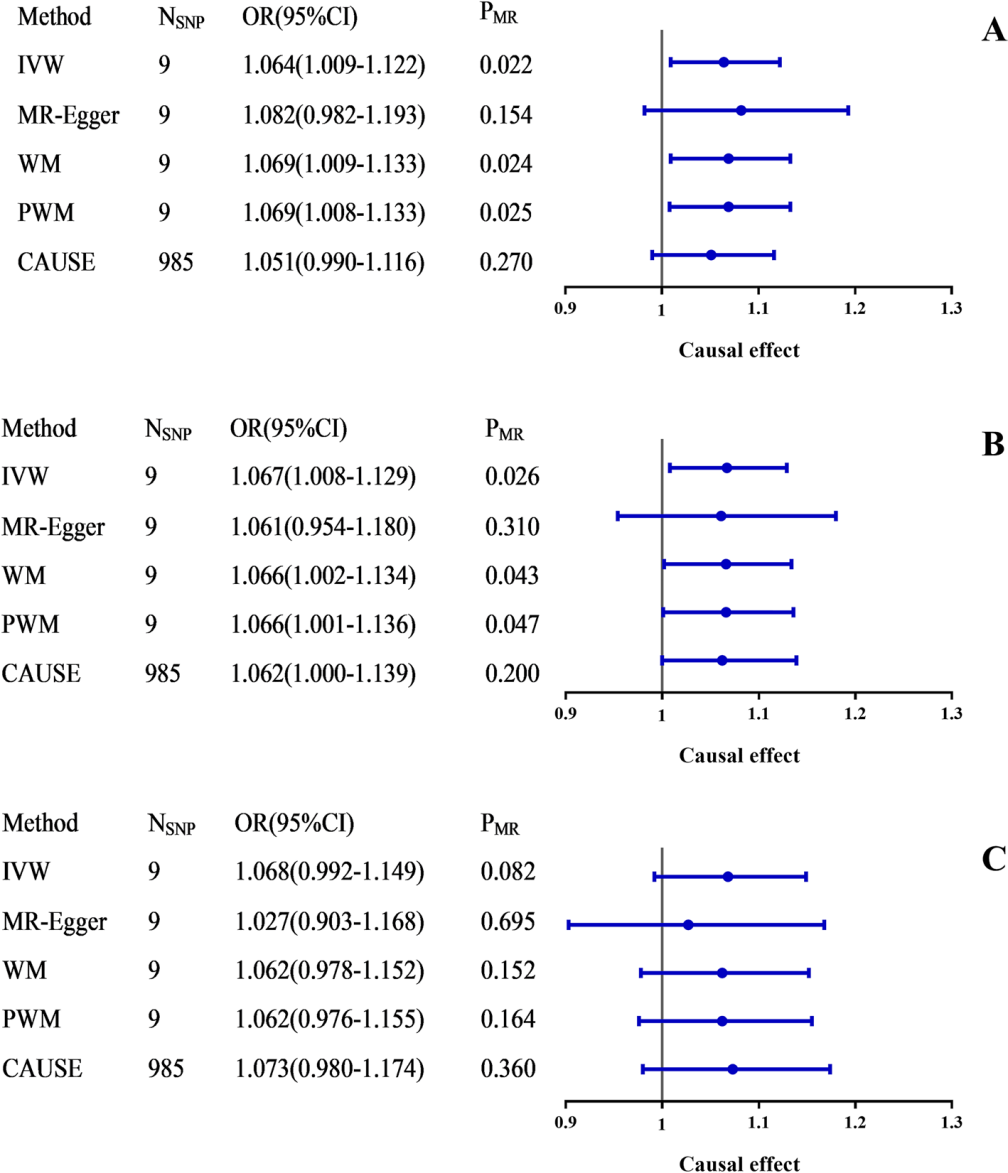
The causal effect of VEGF on the risk of VTE, DVT_PE and DVT estimated using five MR methods. **A**: VEGF to VTE; **B**: VEGF to DVT_PE; **C**: VEGF to DVT. The causal effect from VEGF to VTE, DVT_PE and DVT was expressed as OR per unit. Error bars represent the 95% CIs of the estimates. CAUSE recruited independent instrumental SNPs with GWAS *p*-value < 1 × 10^− 3^. SNP: single-nucleotide polymorphism; GWAS: genome-wide association study; VEGF: vascular endothelial growth factor; VTE: venous thromboembolism; DVT_PE: deep vein thrombosis and pulmonary embolism; DVT: DVT of the lower extremities; IVW: inverse-variance weighted; WM: weighted median; PWM: penalty weighted median; CAUSE: causal analysis using summary effect estimates; OR: odds ratio; CIs: confidence intervals; MR: Mendelian randomization

Figure 2 A-C and Table S3 (supplementary material) showed the MR estimates for VEGF on VTE risk using different methods. MR provided suggestive evidence (three of the five methods with *P* < 0.05) for a causal effect from VEGF to VTE using 9 SNPs. The IVW, WM and PWM estimates showed that individuals with higher VEGF levels may have high risk in VTE development (OR_IVW_ = 1.064, 95% CI, 1.009–1.122, *P*_IVW_ = 0.022) (Fig. 2 A). Besides, the suggestive causal effect from VEGF to VTE is unlikely to be affected by the pleiotropy because of the limited MR-Egger intercept (-0.005) and non-significant results (*P*_intercept_ = 0.683) in the pleiotropy test. CAUSE analyses indicated that the causal model was better than the sharing model (Table S4), but the difference did not reach the threshold of significance (*P*_CAUSE_ = 0.270), which possibly was due to the low power of the VEGF and VTE GWAS. For IVs, no potential outlier SNP was detected by MR-PRESSO.

According to the main IVW analyses, the results of subgroup analyses of the causal effect of VEGF on DVT_PE (OR = 1.067, 95% CI, 1.008–1.129, *P* = 0.026) were consistent with above (Fig. 2B), but no causal association of VEGF with DVT risk (OR = 1.068, 95% CI, 0.992–1.149, *P* = 0.082) was identified (Fig. 2C). The causal inferences were both robust without heterogeneity and horizontal pleiotropy. No potential outlier SNP was detected by MR-PRESSO (Table S3).

No directional pleiotropy was found, as the funnel plots showed no evidence of asymmetry (Figure S1). Please see Table S3 and S4 in supplementary material for detailed results.

### Causal effect of VTE on VEGF via reverse MR

In the reverse MR analyses, a total of 11 SNPs for VTE, 9 SNPs for DVT_PE, and 7 SNPs for DVT were selected and the F-statistics were ranged from 948 to 12,694 for VTE, 958 to 12,821 for DVT_PE, and 1,641 to 29,967 for DVT, respectively. At last, 10 (rs13377102 was excluded for being palindromic with intermediate allele frequencies), 7 (rs13377102 and rs17092456 were excluded) and 5 (rs13377102 and rs11602537 were exclude) independent SNPs were selected as the IVs for VTE, DVT_PE and DVT after harmonizing SNP-exposure and SNP-outcomes, respectively.

**Fig. 3:**
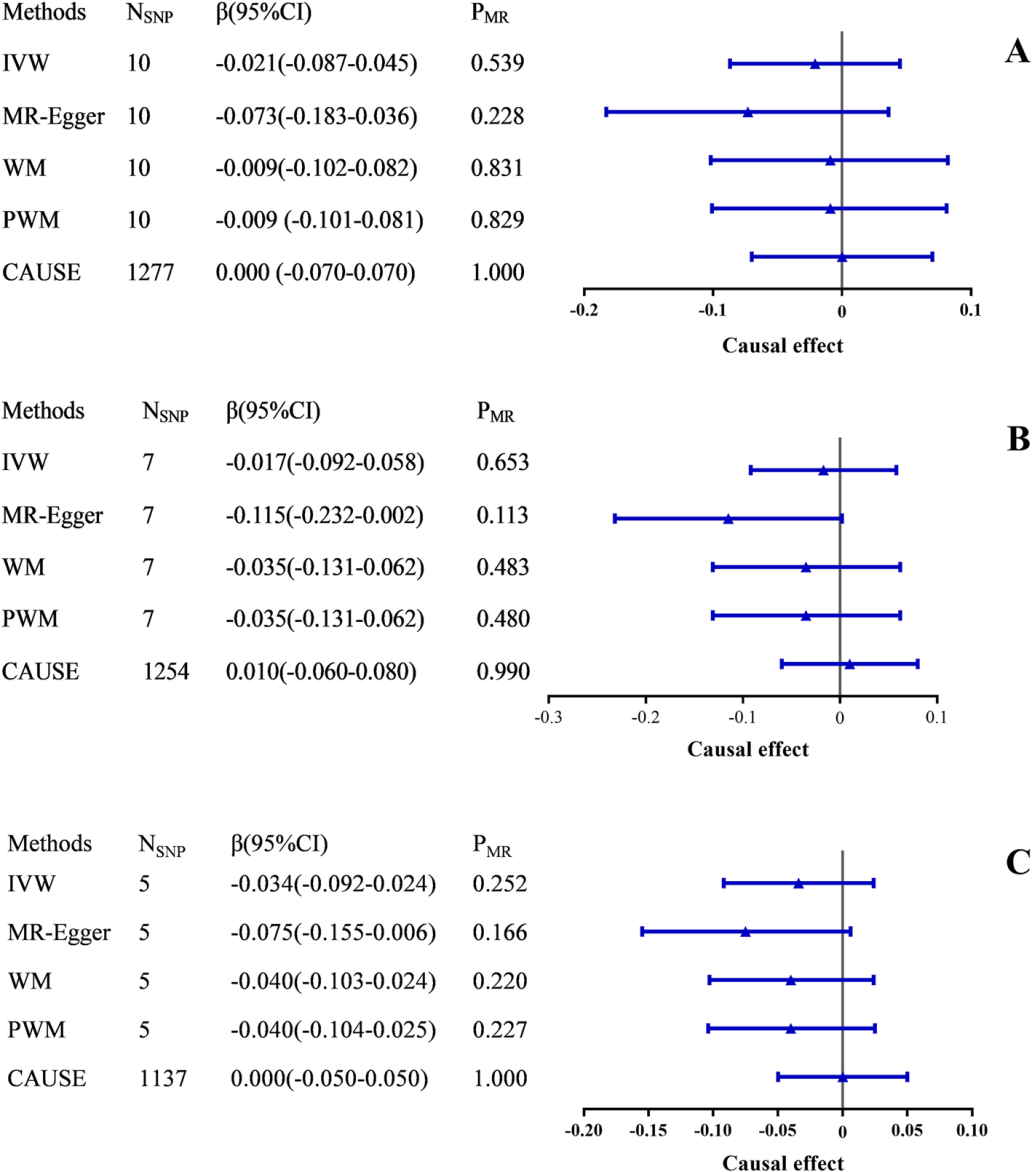
The causal effects of VTE, DVT_PE and DVT on VEGF estimated using five MR methods. **A**: VTE to VEGF; **B**: DVT_PE to VEGF; **C**: DVT to VEGF. The causal effects from VTE, DVT_PE and DVT to VEGF were expressed as β. Error bars represent the 95% CIs of the estimates. CAUSE recruited independent instrumental SNPs with GWAS *p*-value < 1 × 10^− 3^. SNP: single-nucleotide polymorphism; GWAS: genome-wide association study; VEGF: vascular endothelial growth factor; VTE: venous thromboembolism; DVT_PE: deep vein thrombosis and pulmonary embolism; DVT: deep vein thrombosis; IVW: inverse-variance weighted; WM: weighted median; PWM: penalty weighted median; CAUSE: causal analysis using summary effect estimates; CIs: confidence intervals; MR: Mendelian randomization

All five methods in reverse MR analyses consistently suggested no significant association of genetically instrumented VTE, DVT_PE and DVT with VEGF (IVW: β _VTE_ = -0.021; 95% CI, -0.087-0.045; *P* = 0.539; β _DVT_PE_ = -0.017; 95% CI, -0.092-0.058; *P* = 0.653; β _DVT_ = -0.034; 95% CI, -0.092-0.024; *P* = 0.252) (Fig. 3 A-C, Table S5 and Table S6). There were no evidence of heterogeneity between IV estimates with IVW methods from individual SNPs (VTE: *Q*_pval = 0.660, *I*^*2*^ = 0.000; DVT_PE: *Q*_pval = 0.281, *I*^*2*^ = 0.194; DVT: *Q*_pval = 0.426, *I*^*2*^ = 0.000) and no pleiotropy effect (VTE: intercept = 0.014, *P*_intercept_ = 0.274; DVT_PE: intercept = 0.033, *P*_intercept_ = 0.103; DVT: intercept = 0.020, *P*_intercept_ = 0.245). No potential outlier SNP was detected by MR-PRESSO. No directional pleiotropy was found, as the funnel plots showed no evidence of asymmetry (Figure S2).

### LDSC regression analyses

The total heritability of VEGF (3.5%, Mean Chi-square = 1.020) and VTE (1.3%, Mean Chi-square = 1.091) was relatively small (Table 2). No result of genetic correlation was obtained from VEGF with VTE, as genetic correlation estimates for two traits with small heritability are generally too noisy to report [45].

**Table 2:** Heritability and genetic correlations of VEGF and VTE

	Heritability				Genetic correlation			
Traits	h^2^ (SE)	Mean Chi-square	Intercept	Ratio	rg(se)	*P*	h^2^_obs(se)	h^2^_int(se)
VEGF	0.035 (0.029)	1.020	1.009	0.454	-	-	0.012(0.003)	1.038(0.011)
VTE	0.013 (0.004)	1.091	1.034	0.371				

*VEGF* Vascular endothelial growth factor, *VTE* Venous thromboembolism

## Discussion

VEGF plays an important role in impacting various physiological of cancer and disease processes, but its function in the formation and progression of VTE remains unclear. Benefitting from the large sample-based GWAS results and less-bias MR approaches, we found that VEGF was a suggestive risk factor for the risks of VTE or DVT_PE, which was not reported by previous literatures.

It is well known that blood flow change, hyper-coagulable state and vessel wall damage are three critical factors for the pathogenesis of thrombosis [47]. VEGF is closely associated with the vascular endothelial system and is a specific vascular permeability factor and chemotactic factor [48]. Previous studies have shown that VEGF plays an important role in the formation process of deep venous thrombosis (DVT), a common complication in patients with different diseases (cancer, Behçet’s disease, etc.) [49–51]. However, another study identified that VEGF did not affect the coagulation function in colorectal cancer patients not complicated by VTE [52]. The possibility that specific VEGF-SNPs may be related to the development of VTE was raised from a comparative study in 2006 [53], indicating a difference in the distribution of VEGF-A + 936 C/T between cancer patients who developed or did not develop VTE (OR = 2.3, 95% CI, 0.9–5.7). In addition, a single-center clinical trial showed that the circulating level of VEGF-D was moderately correlated with the mean pulmonary artery pressure (*r* = 0.481, *P* = 0.010), and a cutoff of 370.1 pg/ml for VEGF-D had relatively high sensitivity (91.4%) and specificity (67.0%) in the intermediate and high risk PE patients [54]. Furthermore, several observational studies also highlighted the role of VEGF as an inflammatory marker in thrombosis [12, 13]. Meanwhile, according the main MR estimates from IVW analyses of this study, we quantified that one SD increase in VEGF level may increase 6.4% probability for VTE development and 6.7% probability for DVT_PE development, which were consistent with above observational studies to some extent and further strengthened the potential evidence that VEGF is a suggestive causal risk factor for VTE (independent of cancer or other relative diseases), suggesting that cancer or relative diseases are not likely mediator or confounder of VEGF-VTE relationship. We did not consider the negative results from CAUSE analyses, as CAUSE has a little lower power than other MR methods (i.e. IVW and WM) when there was a assured causal effect of VEGF on VTE and no shared factor [32]. As an aggregate illness that includes DVT, PE, or both, DVT with PE is the most severe type and has a higher diagnostic accuracy than DVT alone [55]. Replication analysis using DVT of lower extremities and pulmonary embolism summary data in our study exhibited consistent results with that of VTE. Unexpectedly, we failed to identify the causal effect of VEGF on DVT of lower extremities in replication analyses. Observational studies indicated that circulating level of VEGF was significantly higher in PE patients than that of healthy controls [56], and plasma level of VEGF-D can be used as a biomarker for thrombus burden assessment in patients with acute PE [54]. The significance influence of VEGF on DVT of lower extremities and pulmonary embolism may hint that the key potential mechanisms underlying the link between VEGF and VTE lying in PE other than DVT alone. Potential mechanisms could be related to hypoxia, low PH or inflammatory cytokines, but not all of the mechanisms have been entirely understood [12, 20]. Taken together, all of the above results are consistent with those of our MR study, which strengthens the reliability of the findings.

Additionally, the results of this study provided genetic evidence, which is less susceptible to confounders and reverse causality bias, for the causal inference of VEGF and VTE. Currently, the diagnosis of VTE relies on a sequential work-up using a combination of a clinical score (e.g., Wells score), D-dimer testing, and imaging (ultrasonography for a suspected DVT, CT scan or ventilation-perfusion scintigraphy for a suspected PE when required) [57]. VTE can be ruled out in patients with both lower clinical probability and the normal D-dimer limits, but patients with higher clinical probability or elevated D-dimer concentration require imaging test to confirm the diagnosis [57, 58]. However, D-dimer test has a high sensitivity of 95% but a low specificity for the diagnosis of VTE [59], which may be influenced physiologically by age, cancer, infection or other inflammatory states, even differences in quantitative, enzyme-linked, immunosorbent assays methods [57]. The aforementioned clinical way to detect D-dimer is easy and efficient, which is of great significance in the diagnosis of DVT and evaluation of the prognosis but has little significance for prevention. A study indicated that VEGF levels in the thrombosis group after operation were closely related to the D-dimer and fibrinogen content [51]. All of the above evidence suggests that the results of MR are not only consistent with other forms of evidence but are a key complement to current forms of evidence for diagnosis and prevention of VTE.

Previous study also indicated that the formation of DVT may stimulate the expression of VEGF [14]. Disappointingly, all analytical methods found no evidence of a causal effect of VTE as well as DVT_PE and DVT on VEGF. Differences in findings between the reverse MR and observational studies may have several reasons. First, down-regulation of VEGF appeared in the early stage of thrombosis, and up-regulation of VEGF can promote the organization and recanalization of thrombus [51], suggesting that VEGF levels may vary with different stages of diseases and even be modified by different drugs [11]. Moreover, in addition to cancer-related and hospitalization-related VTE, unprovoked-VTE accounts for 20–30% of the disease burden of VTE [3]. Patients with unprovoked VTE are younger, which is consistent with the estimation of the higher attributable risk for genetic factors in younger patients, while the attributable risk of some specific genetic conditions in elderly patients is only about 7–22% [3]. Therefore, the age composition of the population may also be one of the potential reasons affecting our results. Although the underlying mechanism between VEGF level and VTE risk is still unclear, our study has provided suggestive genetic evidence for a clinical concern to support the importance of VEGF assessments in monitoring and preventing the risk of VTE, especially DVT complicated by pulmonary embolism.

Although the design of MR study is less susceptible to potential confounders and inverse causality, limitations exist. First, our study focused on circulating levels of VEGF and the conclusion cannot be generalized to the function of intracellular levels of VEGF on VTE risk; second, the summary GWAS data used in this study were derived from European population, so our conclusions may not generalize to other ethnic populations; third, the VEGF family includes multiple subtypes (VEGF-A, VEGF-D, etc.) [60], and limited by current knowledge and the inability to obtain both individual-level and summary data for GWAS of VEGF-subtypes and risk factors of VTE to assess potential genetic correlations, we cannot explore other exposures and cannot rule out the possibility of pleiotropy effects. Nonetheless, we performed MR-Egger regression and CAUSE analyses, which were more robust to invalid SNPs and considered the correlated and uncorrelated pleiotropy effects; fourth, the difference in the sample size of GWAS-VEGF and GWAS-VTE may lead to unstable statistical results, and the identified SNPs may exhibit potential weak instrument bias, but this is less likely because the F-statistics for each SNP used was significantly higher than ten. However, small sample size of VEGF could be one of the important reasons for the failure of gene association analysis (LDSC). Therefore, the data of larger samples needs to be discovered and verified. Last but not least, a well conducted MR design, which reasonably satisfies the three key assumptions, usually provides more reliable evidence than a traditional observational study. The MR results should be interpreted cautiously based on the existing evidence of different study designs [61]. Therefore, a clinical trial or large observational study, especially in Asian populations, could be considered to provide definitive evidence.

## Conclusion

Taken together, our findings reported no coheritability between VEGF and VTE. However, this study found a suggestive evidence of causality between VEGF and VTE as well as DVT of the lower extremities and pulmonary embolism, highlighting VEGF as a possible predictor and therapeutic target for VTE (especially DVT complicated by PE) prevention. Since a randomized controlled trial (RCT) is unlikely to be conducted in the short term, the combination of MR and the existing observational evidence can provide relatively reliable evidence for the causal inference of VEGF and VTE and can be used to guide patient care. Raising public awareness and surveillance of VTE and the potential risk factors is an equally important public health goal of reducing mortality related to VTE events. Identification of patients at higher risk for VTE may lead to a more targeted preventive treatment of those individuals. In particular, VEGF levels can be modified by drugs [11], and more attention should be given to patients taking related drugs to prevent or detect the risk of thrombosis early. Especially for young patients with a low awareness rate, regular examinations should be considered. Additionally, MR studies using individual-level statistics may be beneficial to elucidate the potential non-linear relation between VEGF level and VTE risk.

## Supplementary Information

**Additional file 1: Table S1.** SNPs for VEGF in the forward MR analyses:Harmonized Data (*r*^2^< 0.01). SNP: single-nucleotide polymorphism;CHR: chromosome; POS: position; OA: other_allele; EA: effect_allele; SE: standard error; VEGF: vascular endothelialgrowth factor; VTE: venous thromboembolism; MR: Mendelian randomization; MAF: minor allele frequency; R^2^:variance for each SNP, *R*^2^ = 2×MAF× (1-MAF) × Beta^2^; F-statistic = R^2^ × (N-2)/(1-R^2^), N: the number of individualsin the exposure GWAS. ^**$**^:SNPs were excluded after performing harmonizing procedure. *: DVT of the lower extremities and pulmonary embolism. ^**#**^: DVT of the lower extremities. **Table S2.** SNPs for VTE in the reverse MR analyses: Harmonized Data(*r*^2^ < 0.001). SNP: single-nucleotide polymorphism; SE: standard error; VEGF: vascular endothelial growth factor; VTE: venous thromboembolism; MR: Mendelian randomization; MAF: minor allele frequency; R^2^:variance for each SNP, *R*^2^ = 2×MAF× (1-MAF) × Beta^2^; F-statistic = R^2^ × (N-2) / (1-R^2^), N: the number of individuals in the exposure GWAS. ^**$**^: SNPs were excluded after performing harmonizing procedure. *: DVT of the lower extremities and pulmonary embolism. ^**#**^:DVT of the lower extremities. **Table S3.**Causal associations of VEGF with risks of VTE, DVT_PE and DVT by forward MR analyses. SNP: single-nucleotide polymorphism; SE: standard error; VEGF: vascular endothelial growth factor;VTE: venous thromboembolism; IVW: inverse-variance weighted; WM: weighted median; PWM:penalty weighted median; CAUSE: causal analysis using summary effect estimates; MR-PRESSO: pleiotropy residual sum and outlier; OR: odds ratio; MR: Mendelian randomization; Q_pval: *P*value of the Cochran Q statistic; *I*^*2*^= (Q-df)/Q×100%; *P* < 0.05 were considered statistically significant. *:DVT of the lower extremities and pulmonary embolism. ^**#**^: DVT of the lower extremities. **Table S4.** Causal associations of VTE, DVT_PE and DVT with VEGF via reverse MR. SNP: single-nucleotide polymorphism; VEGF: vascular endothelial growth factor; VTE: venous thromboembolism; IVW: inverse-variance weighted; WM: weighted median; PWM:penalty weighted median; CAUSE: causal analysis using summary effect estimates; MR-PRESSO: pleiotropy residual sum and outlier; MR: Mendelian randomization; Q_pval: *P*value of the Cochran Q statistic; *I*^*2*^= (Q-df)/Q×100%; *P* < 0.05 were considered statistically significant. *:DVT of the lower extremities and pulmonary embolism. ^**#**^: DVT of the lower extremities. **Table S5.** The results of CAUSE analyses via forward MR. CAUSE: causal analysis using summary effect estimates; VEGF: vascular endothelial growth factor; VTE: venous thromboembolism; MR: Mendelian randomization. *: DVT of the lower extremities and pulmonary embolism. ^**#**^: DVT of the lower extremities.**Table S6.** The results of CAUSE analyses via reverse MR.CAUSE: causal analysis using summary effect estimates; VEGF: vascular endothelial growth factor; VTE:venous thromboembolism; MR: Mendelian randomization.*: DVT of the lower extremities and pulmonary embolism. ^**#**^: DVT of the lower extremities. **Figure S1.** MR Funnel plots (VEGF to VTE). VEGF: vascular endothelial growth factor; VTE:venous thromboembolism; MR: Mendelian randomization.**Figure S2.** MR Funnel plots (VTE to VEGF). VEGF: vascular endothelial growth factor; VTE:venous thromboembolism; MR: Mendelian randomization.

## Data Availability

All summary datasets analyzed during the current study are publicly available and all corresponding links are included in this published article and its references.

## Abbreviations

CAUSE: Causal analysis using summary effect estimates
CI: Confidence interval
DVT: Deep vein thrombosis
GWAS: Genome-wide association studies
IVW: Inverse variance weighting
LD: Linkage disequilibrium
LDSC: Linkage disequilibrium score regression
MR: Mendelian randomization
OR: Odds ratio
PWM: Penalty weighted median
PRESSO: Pleiotropy residual sum and outlier
PE: Pulmonary embolism
RCT: Randomized controlled trial
SD: Standard deviation
SNP: Single nucleotide polymorphisms
UVMR: Univariable mendelian randomization
VEGF: Vascular endothelial growth factor
VTE: Venous thromboembolism
WM: Weighted median
