# Supplementary material for: Vascular endothelial growth factor and the risk of venous thromboembolism: a genetic correlation and two-sample Mendelian randomization study
Source: Thromb J. 2022 Nov 8;20:67. doi: 10.1186/s12959-022-00427-6 (PMC9644522; doi:10.1186/s12959-022-00427-6)
Supplement: Supplementary file 1 — Additional file 1: Table S1. SNPs for VEGF in the forward MR analyses:Harmonized Data (r2< 0.01). SNP: single-nucleotide polymorphism;CHR: chromosome; POS: position; OA: other_allele; EA: effect_allele; SE: standard error; VEGF: vascular endothelialgrowth factor; VTE: venous thromboembolism; MR: Mendelian randomization; MAF: minor allele frequency; R2:variance for each SNP, R2 = 2×MAF× (1-MAF) × Beta2; F-statistic = R2 × (N-2)/(1-R2), N: the number of individualsin the exposure GWAS. $:SNPs were excluded after performing harmonizing procedure. *: DVT of the lower extremities and pulmonary embolism. #: DVT of the lower extremities. Table S2. SNPs for VTE in the reverse MR analyses: Harmonized Data(r2 < 0.001). SNP: single-nucleotide polymorphism; SE: standard error; VEGF: vascular endothelial growth factor; VTE: venous thromboembolism; MR: Mendelian randomization; MAF: minor allele frequency; R2:variance for each SNP, R2 = 2×MAF× (1-MAF) × Beta2; F-statistic = R2 × (N-2) / (1-R2), N: the number of individuals in the exposure GWAS. $: SNPs were excluded after performing harmonizing procedure. *: DVT of the lower extremities and pulmonary embolism. #:DVT of the lower extremities. Table S3.Causal associations of VEGF with risks of VTE, DVT_PE and DVT by forward MR analyses. SNP: single-nucleotide polymorphism; SE: standard error; VEGF: vascular endothelial growth factor;VTE: venous thromboembolism; IVW: inverse-variance weighted; WM: weighted median; PWM:penalty weighted median; CAUSE: causal analysis using summary effect estimates; MR-PRESSO: pleiotropy residual sum and outlier; OR: odds ratio; MR: Mendelian randomization; Q_pval: Pvalue of the Cochran Q statistic; I2= (Q-df)/Q×100%; P < 0.05 were considered statistically significant. *:DVT of the lower extremities and pulmonary embolism. #: DVT of the lower extremities. Table S4. Causal associations of VTE, DVT_PE and DVT with VEGF via reverse MR. SNP: single-nucleotide polymorphism; VEGF: vascular endothelial growth [file 12959_2022_427_MOESM1_ESM.docx]

**Supplementary material**

**Table S1** SNPs for VEGF in the forward MR analyses: Harmonized Data (r^2^ < 0.01)

|  |  |  |  |  |  |  |  |  |  |  | Outcome: VTE | | | Outcome: DVT_PE***** | | | Outcome: DVT**^#^** | | |  |
| --- | --- | --- | --- | --- | --- | --- | --- | --- | --- | --- | --- | --- | --- | --- | --- | --- | --- | --- | --- | --- |
| SNP | CHR | POS | OA | EA | MAF | Beta | SE | *P* | R^2^ | F-statistic | Beta | SE | *P* | Beta | SE | *P* | Beta | SE | *P* | |
| rs9381249 | 6 | 43734798 | T | C | 0.0109 | 0.2482 | 0.0397 | 3.09E-10 | 0.001328312 | 291.0080349 | 0.0817 | 0.039 | 0.03619 | 0.0881 | 0.0417 | 0.03453 | 0.0137 | 0.0542 | 0.8004 | |
| rs6920532 | 6 | 43793430 | T | C | 0.8936 | -0.1803 | 0.0267 | 8.68E-12 | 0.006181676 | 1360.90154 | 0.0198 | 0.0259 | 0.444 | 0.0152 | 0.0276 | 0.5831 | 4.00E-04 | 0.0358 | 0.9905 | |
| rs67798973 | 6 | 43882777 | A | G | 0.4433 | -0.1389 | 0.0175 | 1.29E-15 | 0.009522554 | 2103.469975 | 0.0161 | 0.0161 | 0.3196 | 0.0183 | 0.0172 | 0.2888 | 0.0156 | 0.0223 | 0.4858 | |
| rs6921438 | 6 | 43925607 | A | G | 0.4672 | 0.49 | 0.0175 | 2.09E-171 | 0.119533382 | 29703.23692 | -0.0364 | 0.0159 | 0.02232 | -0.0338 | 0.017 | 0.04727 | -0.0295 | 0.0221 | 0.1805 | |
| rs9472183 | 6 | 43940202 | A | G | 0.5109 | 0.1282 | 0.017 | 5.19E-14 | 0.008213715 | 1811.961565 | -0.0057 | 0.0159 | 0.7185 | 0.0034 | 0.017 | 0.8399 | 0.0158 | 0.022 | 0.4741 | |
| rs74675876 | 6 | 43963995 | T | C | 0.0298 | 0.2822 | 0.0366 | 7.62E-15 | 0.004604914 | 1012.170149 | -0.0237 | 0.0359 | 0.5085 | -0.0263 | 0.0383 | 0.4932 | -0.001 | 0.0496 | 0.9838 | |
| rs4507572 | 6 | 44135095 | T | C | 0.4702 | -0.1007 | 0.0171 | 3.34E-09 | 0.005052235 | 1110.991415 | -0.0178 | 0.0159 | 0.2635 | -0.0262 | 0.017 | 0.1235 | -0.042 | 0.022 | 0.05611 | |
| rs41282660 | 6 | 44197006 | A | G | 0.8678 | 0.1613 | 0.0263 | 1.33E-09 | 0.005969663 | 1313.946435 | -0.0067 | 0.0235 | 0.7742 | -0.0138 | 0.0251 | 0.583 | -0.0082 | 0.0325 | 0.801 | |
| rs34881325 | 9 | 2622134 | T | C | 0.5736 | 0.1082 | 0.0189 | 1.04E-08 | 0.005726785 | 1260.180004 | -0.0012 | 0.0167 | 0.9418 | -0.0017 | 0.0178 | 0.9251 | 9.00E-04 | 0.0231 | 0.968 | |
| rs7030781**^$^** | 9 | 2686273 | A | T | 0.5815 | -0.1368 | 0.0173 | 2.57E-15 | 0.009108511 | 2011.169813 | 0.0000 | 0.0162 | 0.9988 | -0.0142 | 0.0173 | 0.4103 | -0.0131 | 0.0224 | 0.5589 | |
| rs10761731**^$^** | 10 | 65027610 | A | T | 0.5676 | 0.1187 | 0.0174 | 1.01E-11 | 0.006916072 | 1523.705449 | 0.0038 | 0.0163 | 0.8139 | -0.0106 | 0.0174 | 0.5437 | -0.0154 | 0.0226 | 0.494 | |

SNP: single-nucleotide polymorphism; CHR: chromosome; POS: position; OA: other_allele; EA: effect_allele; SE: standard error; VEGF: vascular endothelial growth factor; VTE: venous thromboembolism; MR: Mendelian randomization; MAF: minor allele frequency; R^2^: variance for each SNP, R^2^ = 2×MAF× (1-MAF) × Beta^2^; F-statistic = R^2^ × (N-2)/(1-R^2^), N: the number of individuals in the exposure GWAS.

**^$^**: SNPs were excluded after performing harmonizing procedure.

*****: DVT of the lower extremities and pulmonary embolism.

**^#^**: DVT of the lower extremities.

**Table S2** SNPs for VTE in the reverse MR analyses: Harmonized Data (r^2^ < 0.001)

|  |  |  |  |  |  |  |  |  |  |  |  | Outcome: VEGF | | |
| --- | --- | --- | --- | --- | --- | --- | --- | --- | --- | --- | --- | --- | --- | --- |
| SNP | Exposure | CHR | POS | OA | EA | MAF | Beta | SE | *P* | R^2^ | F-statistic | Beta | SE | *P* |
| rs6025 | VTE | 1 | 169549811 | C | T | 0.02026 | 1.1753 | 0.0624 | 3.52E-79 | 0.054838 | 12694.01 | 0.1294 | 0.0644 | 0.04347 |
| rs2066865 | VTE | 4 | 154604124 | G | A | 0.3021 | 0.2171 | 0.0174 | 6.92E-36 | 0.019874 | 4436.488 | -0.0118 | 0.0183 | 0.5279 |
| rs3756011 | VTE | 4 | 186285095 | C | A | 0.4274 | 0.2044 | 0.016 | 2.89E-37 | 0.020449 | 4567.496 | -0.003 | 0.0172 | 0.8639 |
| rs62350309 | VTE | 4 | 186356512 | A | G | 0.1031 | -0.164 | 0.0263 | 4.71E-10 | 0.004974 | 1093.738 | 0.0181 | 0.0294 | 0.5091 |
| rs628094 | VTE | 9 | 133001900 | G | A | 0.6825 | 0.1039 | 0.0171 | 1.13E-09 | 0.004679 | 1028.422 | 0.0092 | 0.0182 | 0.6124 |
| rs149244513 | VTE | 9 | 133183871 | G | A | 0.03032 | 0.2709 | 0.0473 | 1.05E-08 | 0.004315 | 948.2247 | -0.026 | 0.0551 | 0.6205 |
| rs13377102**^$^** | VTE | 10 | 69453630 | T | A | 0.1089 | -0.1836 | 0.0257 | 9.22E-13 | 0.006542 | 1440.813 | 0.0165 | 0.0281 | 0.5899 |
| rs5896 | VTE | 11 | 46723453 | C | T | 0.2217 | 0.1129 | 0.0202 | 2.44E-08 | 0.004399 | 966.6575 | 0.0013 | 0.0199 | 0.949 |
| rs117716477 | VTE | 12 | 103847180 | C | A | 0.0165 | 0.4855 | 0.0659 | 1.76E-13 | 0.00765 | 1686.667 | -0.0487 | 0.059 | 0.4318 |
| rs2885055 | VTE | 19 | 10618421 | G | A | 0.8221 | 0.1381 | 0.0208 | 3.30E-11 | 0.005579 | 1227.368 | -0.0232 | 0.0219 | 0.2934 |
| rs6060308 | VTE | 20 | 35206575 | G | A | 0.3178 | 0.1143 | 0.0171 | 2.17E-11 | 0.005665 | 1246.473 | 0.0011 | 0.0182 | 0.9536 |
| rs1894692 | DVT_PE***** | 1 | 169498416 | G | A | 0.9798 | -1.1826 | 0.0668 | 4.41E-70 | 0.05536 | 12821.99 | -0.1345 | 0.0633 | 0.03219 |
| rs141867395 | DVT_PE | 1 | 169501496 | C | T | 0.08629 | 0.1663 | 0.0302 | 3.50E-08 | 0.004361 | 958.3163 | -0.0251 | 0.0309 | 0.4169 |
| rs2066865 | DVT_PE | 4 | 154604124 | G | A | 0.3021 | 0.2203 | 0.0185 | 1.40E-32 | 0.020465 | 4570.99 | -0.0118 | 0.0183 | 0.5279 |
| rs3756011 | DVT_PE | 4 | 186285095 | C | A | 0.4274 | 0.2241 | 0.0171 | 3.87E-39 | 0.024581 | 5513.607 | -0.003 | 0.0172 | 0.8639 |
| rs62350309 | DVT_PE | 4 | 186356512 | A | G | 0.1031 | -0.1649 | 0.0281 | 4.38E-09 | 0.005029 | 1105.837 | 0.0181 | 0.0294 | 0.5091 |
| rs13377102**^$^** | DVT_PE | 10 | 69453630 | T | A | 0.1089 | -0.187 | 0.0274 | 9.43E-12 | 0.006787 | 1495.039 | 0.0165 | 0.0281 | 0.5899 |
| rs117716477 | DVT_PE | 12 | 103847180 | C | A | 0.0165 | 0.539 | 0.0708 | 2.73E-14 | 0.009429 | 2082.609 | -0.0487 | 0.059 | 0.4318 |
| rs1560711 | DVT_PE | 19 | 10631611 | C | T | 0.8238 | 0.1429 | 0.0223 | 1.47E-10 | 0.005928 | 1304.764 | -0.0221 | 0.0219 | 0.3127 |
| rs17092456**^$^** | DVT_PE | 20 | 35208389 | G | C | 0.1387 | 0.1608 | 0.0247 | 8.26E-11 | 0.006178 | 1360.04 | -0.0188 | 0.0256 | 0.4646 |
| rs6025 | DVT**^#^** | 1 | 169549811 | C | T | 0.02026 | 1.742 | 0.0906 | 1.86E-82 | 0.120469 | 29967.68 | 0.1294 | 0.0644 | 0.04347 |
| rs2066865 | DVT | 4 | 154604124 | G | A | 0.3021 | 0.19 | 0.024 | 2.12E-15 | 0.015222 | 3381.975 | -0.0118 | 0.0183 | 0.5279 |
| rs3756011 | DVT | 4 | 186285095 | C | A | 0.4274 | 0.2118 | 0.0222 | 1.40E-21 | 0.021957 | 4911.76 | -0.003 | 0.0172 | 0.8639 |
| rs13377102**^$^** | DVT | 10 | 69453630 | T | A | 0.1089 | -0.1959 | 0.0356 | 3.63E-08 | 0.007448 | 1641.827 | 0.0165 | 0.0281 | 0.5899 |
| rs11602537**^$^** | DVT | 11 | 46709089 | C | G | 0.2226 | 0.1644 | 0.028 | 4.40E-09 | 0.009354 | 2065.915 | -0.0018 | 0.0199 | 0.9303 |
| rs117716477 | DVT | 12 | 103847180 | C | A | 0.0165 | 0.5204 | 0.0906 | 9.27E-09 | 0.008789 | 1940.101 | -0.0487 | 0.059 | 0.4318 |
| rs6060308 | DVT | 20 | 35206575 | G | A | 0.3178 | 0.1567 | 0.0236 | 3.40E-11 | 0.010647 | 2354.561 | 0.0011 | 0.0182 | 0.9536 |

SNP: single-nucleotide polymorphism; SE: standard error; VEGF: vascular endothelial growth factor; VTE: venous thromboembolism; MR: Mendelian randomization; MAF: minor allele frequency; R^2^: variance for each SNP, R^2^ = 2×MAF× (1-MAF) × Beta^2^; F-statistic = R^2^ × (N-2) / (1-R^2^), N: the number of individuals in the exposure GWAS.

**^$^**: SNPs were excluded after performing harmonizing procedure.

*****: DVT of the lower extremities and pulmonary embolism.

**^#^**: DVT of the lower extremities.

**Table S3** Causal associations of VEGF with risks of VTE, DVT_PE and DVT by forward MR analyses

| Exposure | Outcome | nSNPs | OR (95%CI) | Beta (SE) | *P* | Q_pval (*I^2^*) | Intercept (*P*) | RSSobs | Global *P* |
| --- | --- | --- | --- | --- | --- | --- | --- | --- | --- |
| VEGF | VTE |  |  |  |  |  |  |  |  |
|  | IVW | 9 | 1.064(1.009-1.122) | 0.062(0.027) | 0.022 | 0.446(0.000) |  |  |  |
|  | MR-Egger | 9 | 1.082(0.982-1.193) | 0.079(0.049) | 0.154 |  | -0.005(0.683) |  |  |
|  | WM | 9 | 1.069(1.009-1.133) | 0.067(0.029) | 0.024 |  |  |  |  |
|  | PWM | 9 | 1.069(1.008-1.133) | 0.067(0.029) | 0.025 |  |  |  |  |
|  | CAUSE | 985 | 1.051(0.990-1.116) | 0.050(0.045) | 0.270 |  |  |  |  |
|  | MR-PRESSO | 9 | 0.952(0.905-1.000) | -0.049(0.025) | 0.083 |  |  | 14.196 | 0.476 |
| VEGF | DVT_PE***** |  |  |  |  |  |  |  |  |
|  | IVW | 9 | 1.067(1.008-1.129) | 0.065(0.029) | 0.026 | 0.430(0.005) |  |  |  |
|  | MR-Egger | 9 | 1.061(0.954-1.180) | 0.059(0.054) | 0.310 |  | 0.002(0.908) |  |  |
|  | WM | 9 | 1.066(1.002-1.134) | 0.064(0.032) | 0.043 |  |  |  |  |
|  | PWM | 9 | 1.066(1.001-1.136) | 0.064(0.032) | 0.047 |  |  |  |  |
|  | CAUSE | 985 | 1.062(1.000-1.139) | 0.060(0.047) | 0.200 |  |  |  |  |
|  | MR-PRESSO | 9 | 0.955(0.893-1.017) | -0.046(0.033) | 0.199 |  |  | 15.271 | 0.349 |
| VEGF | DVT**^#^** |  |  |  |  |  |  |  |  |
|  | IVW | 9 | 1.068(0.992-1.149) | 0.065(0.038) | 0.082 | 0.897(0.000) |  |  |  |
|  | MR-Egger | 9 | 1.027(0.903-1.168) | 0.027(0.066) | 0.695 |  | 0.011(0.499) |  |  |
|  | WM | 9 | 1.062(0.978-1.152) | 0.060(0.042) | 0.152 |  |  |  |  |
|  | PWM | 9 | 1.062(0.976-1.155) | 0.060(0.043) | 0.164 |  |  |  |  |
|  | CAUSE | 985 | 1.073(0.980-1.174) | 0.070(0.076) | 0.360 |  |  |  |  |
|  | MR-PRESSO | 9 | 0.973(0.916-1.030) | -0.027(0.030) | 0.379 |  |  | 10.674 | 0.685 |

SNP: single-nucleotide polymorphism; SE: standard error; VEGF: vascular endothelial growth factor; VTE: venous thromboembolism; IVW: inverse-variance weighted; WM: weighted median; PWM: penalty weighted median; CAUSE: causal analysis using summary effect estimates; MR-PRESSO: pleiotropy residual sum and outlier; OR: odds ratio; MR: Mendelian randomization; Q_pval: *P* value of the Cochran Q statistic; *I^2^* = (Q-df)/Q×100%; *P* < 0.05 were considered statistically significant.

*****: DVT of the lower extremities and pulmonary embolism.

**^#^**: DVT of the lower extremities.

**Table S4** The results of CAUSE analyses via forward MR

| model 1 | model 2 | VEGF to VTE | | | | VEGF to DVT_PE***** | | | | VEGF to DVT**^#^** | | | |
| --- | --- | --- | --- | --- | --- | --- | --- | --- | --- | --- | --- | --- | --- |
|  |  | delta_elpd | se _delta_elpd | z | *P* | delta_elpd | se _delta_elpd | z | *P* | delta_elpd | se _delta_elpd | z | *P* |
| null | sharing | 0.250 | 0.300 | 0.820 | 0.790 | 0.200 | 0.270 | 0.740 | 0.770 | 0.250 | 0.390 | 0.650 | 0.740 |
| null | causal | -0.630 | 1.600 | -0.400 | 0.350 | -1.100 | 1.700 | -0.630 | 0.260 | -0.055 | 1.100 | -0.049 | 0.480 |
| sharing | causal | -0.880 | 1.400 | -0.620 | 0.270 | -1.300 | 1.600 | -0.830 | 0.200 | -0.310 | 0.850 | -0.360 | 0.360 |

CAUSE: causal analysis using summary effect estimates; VEGF: vascular endothelial growth factor; VTE: venous thromboembolism; MR: Mendelian randomization.

*****: DVT of the lower extremities and pulmonary embolism.

**^#^**: DVT of the lower extremities.

**Table S5** Causal associations of VTE, DVT_PE and DVT with VEGF via reverse MR

| Exposure | Outcome | nSNPs | β (95%CI) | *P* | Q_pval(*I^2^*) | Intercept(*P*) | RSSobs | Global *P* |
| --- | --- | --- | --- | --- | --- | --- | --- | --- |
| VTE | VEGF |  |  |  |  |  |  |  |
| IVW |  | 10 | -0.021(-0.087-0.045) | 0.539 | 0.660(0.000) |  |  |  |
| MR-Egger |  | 10 | -0.073(-0.183-0.036) | 0.228 |  | 0.014(0.274) |  |  |
| WM |  | 10 | -0.009(-0.102-0.082) | 0.831 |  |  |  |  |
| PWM |  | 10 | -0.009 (-0.101-0.081) | 0.829 |  |  |  |  |
| CAUSE |  | 1277 | 0.000 (-0.070-0.070) | 1.000 |  |  |  |  |
| MR-PRESSO |  | 10 | 0.008 (-0.048-0.061) | 0.780 |  |  | 13.021 | 0.480 |
| DVT_PE***** | VEGF |  |  |  |  |  |  |  |
| IVW |  | 7 | -0.017(-0.092-0.058) | 0.653 | 0.281(0.194) |  |  |  |
| MR-Egger |  | 7 | -0.115(-0.232-0.002) | 0.113 |  | 0.033(0.103) |  |  |
| WM |  | 7 | -0.035(-0.131-0.062) | 0.483 |  |  |  |  |
| PWM |  | 7 | -0.035(-0.131-0.062) | 0.480 |  |  |  |  |
| CAUSE |  | 1254 | 0.010(-0.060-0.080) | 0.990 |  |  |  |  |
| MR-PRESSO |  | 7 | 0.009(-0.070-0.083) | 0.822 |  |  | 15.517 | 0.232 |
| DVT**^#^** | VEGF |  |  |  |  |  |  |  |
| IVW |  | 5 | -0.034(-0.092-0.024) | 0.252 | 0.426(0.000) |  |  |  |
| MR-Egger |  | 5 | -0.075(-0.155-0.006) | 0.166 |  | 0.020(0.245) |  |  |
| WM |  | 5 | -0.040(-0.103-0.024) | 0.220 |  |  |  |  |
| PWM |  | 5 | -0.040(-0.104-0.025) | 0.227 |  |  |  |  |
| CAUSE |  | 1137 | 0.000(-0.050-0.050) | 1.000 |  |  |  |  |
| MR-PRESSO |  | 5 | 0.034(-0.024-0.090) | 0.308 |  |  | 12.407 | 0.375 |

SNP: single-nucleotide polymorphism; VEGF: vascular endothelial growth factor; VTE: venous thromboembolism; IVW: inverse-variance weighted; WM: weighted median; PWM: penalty weighted median; CAUSE: causal analysis using summary effect estimates; MR-PRESSO: pleiotropy residual sum and outlier; MR: Mendelian randomization; Q_pval: *P* value of the Cochran Q statistic; *I^2^* = (Q-df)/Q×100%; *P* < 0.05 were considered statistically significant.

*****: DVT of the lower extremities and pulmonary embolism.

**^#^**: DVT of the lower extremities.

**Table S6** The results of CAUSE analyses via reverse MR

| model 1 | model 2 | VTE to VEGF | | | | DVT_PE***** to VEGF | | | | DVT**^#^** to VEGF | | | |
| --- | --- | --- | --- | --- | --- | --- | --- | --- | --- | --- | --- | --- | --- |
|  |  | delta_elpd | se _delta_elpd | z | *P* | delta_elpd | se _delta_elpd | z | *P* | delta_elpd | se _delta_elpd | z | *P* |
| null | sharing | 0.190 | 0.670 | 0.280 | 0.610 | 0.380 | 0.310 | 1.200 | 0.890 | 0.400 | 0.094 | 4.200 | 1.000 |
| null | causal | 0.930 | 0.770 | 1.200 | 0.890 | 1.200 | 0.590 | 2.100 | 0.980 | 1.300 | 0.250 | 5.200 | 1.000 |
| sharing | causal | 0.750 | 0.210 | 3.500 | 1.000 | 0.860 | 0.360 | 2.400 | 0.990 | 0.890 | 0.190 | 4.600 | 1.000 |

CAUSE: causal analysis using summary effect estimates; VEGF: vascular endothelial growth factor; VTE: venous thromboembolism; MR: Mendelian randomization.

*****: DVT of the lower extremities and pulmonary embolism.

**^#^**: DVT of the lower extremities.


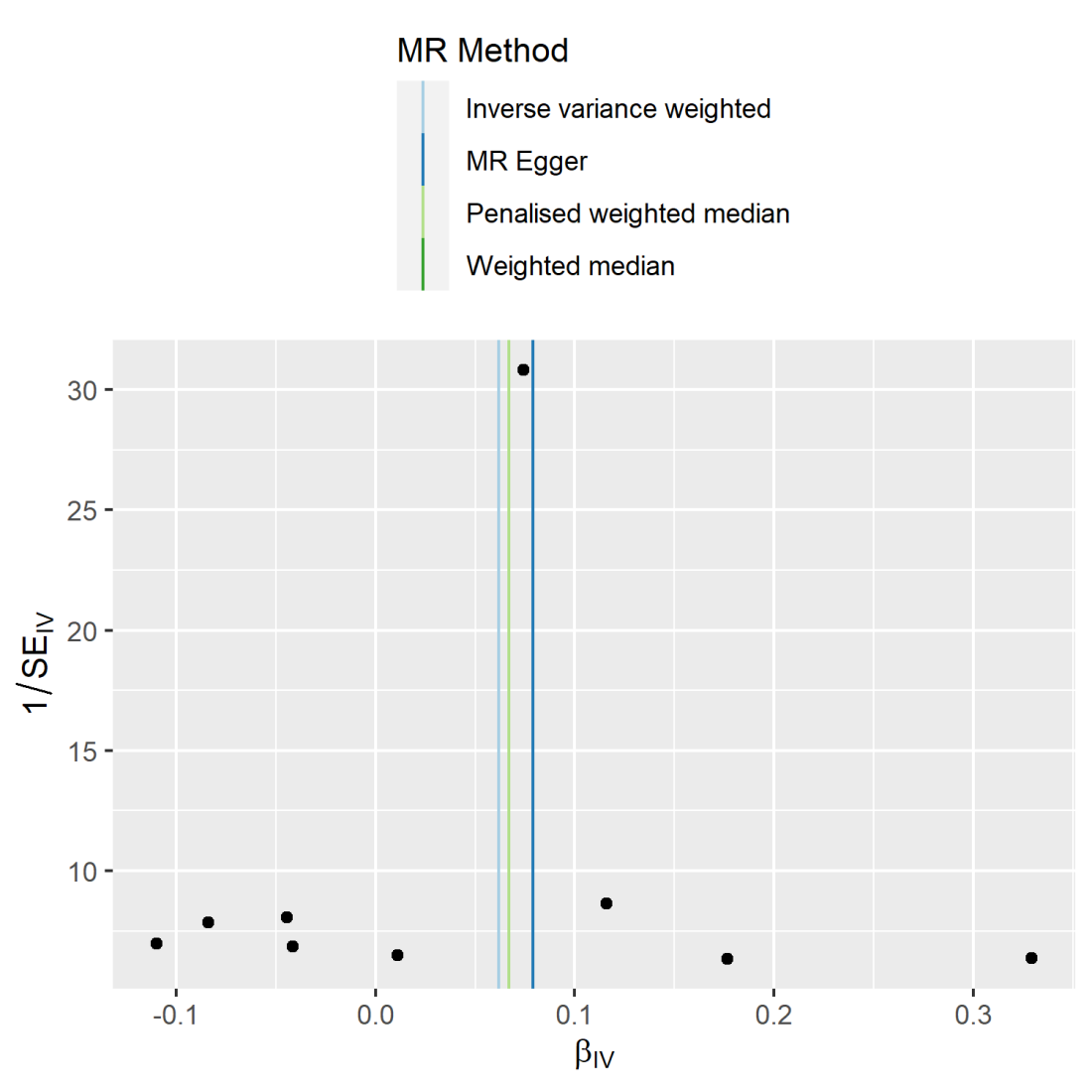


**Figure S1** MR Funnel plots (VEGF to VTE)

VEGF: vascular endothelial growth factor; VTE: venous thromboembolism; MR: Mendelian randomization.


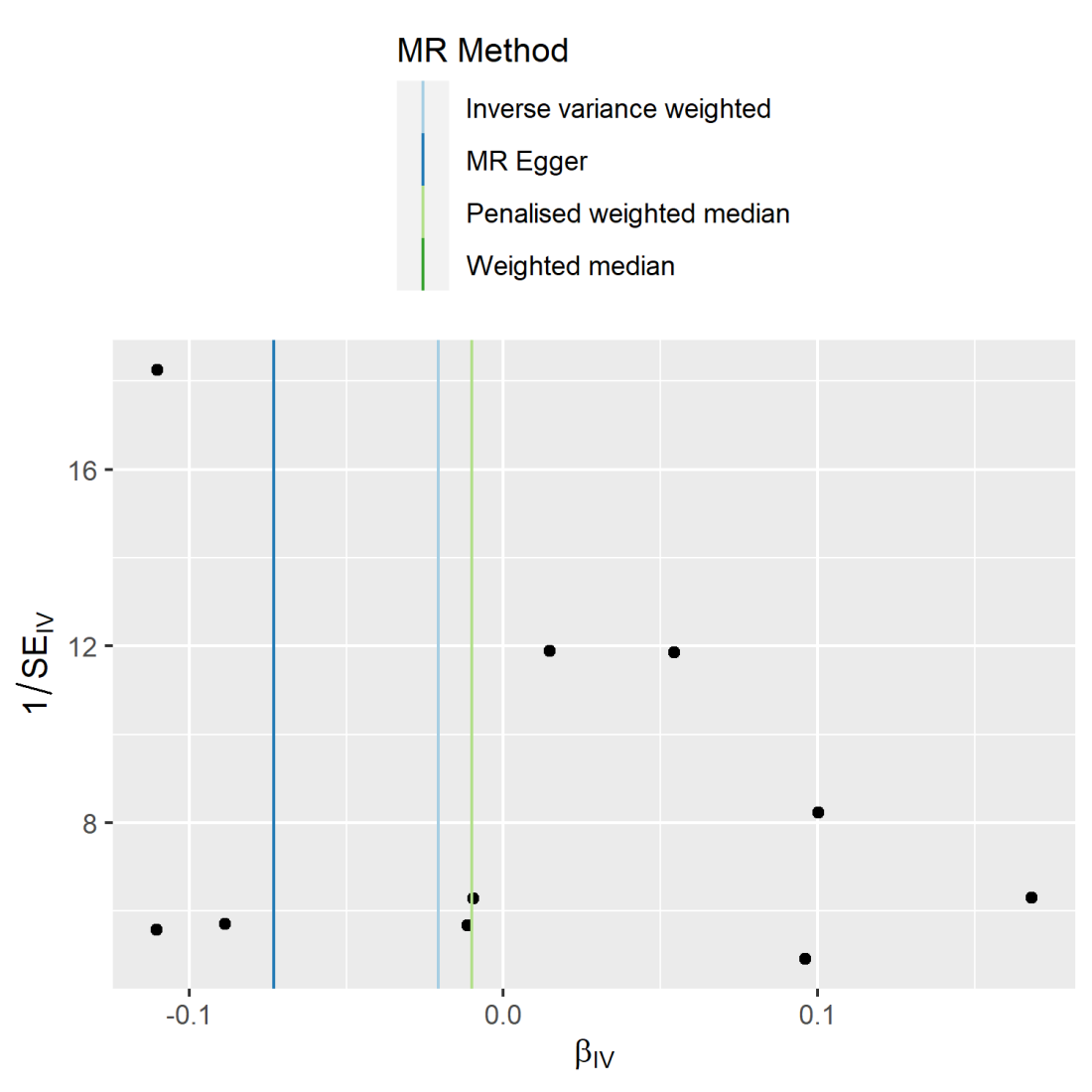


**Figure S2** MR Funnel plots (VTE to VEGF).

VEGF: vascular endothelial growth factor; VTE: venous thromboembolism; MR: Mendelian randomization.
